# Excellent Dynamic Non‐Wetting Performance Induced by Asymmetric Structure at Low Temperatures: Retraction Actuation and Nucleation Inhibition

**DOI:** 10.1002/advs.202500590

**Published:** 2025-02-28

**Authors:** Jiawei Jiang, Yizhou Shen, Yangjiangshan Xu, Zhen Wang, Senyun Liu, Yanyan Lin, Jie Tao, Zhong Chen

**Affiliations:** ^1^ State Key Laboratory of Mechanics and Control for Aerospace Structures Nanjing University of Aeronautics and Astronautics No. 29 Yudao Street Nanjing 210016 P. R. China; ^2^ Beijing Blue Sky Innovation Center for Frontier Science Yard 11, anningzhuang Road, Haidian District Beijing 100080 P. R. China; ^3^ Key Laboratory of Icing and Anti/De‐icing China Aerodynamics Research and Development Center 6 Erhuan South Rd. Mianyang 621000 P. R. China; ^4^ School of Materials Science and Engineering Nanjing Institute of Technology Nanjing 211167 P. R. China; ^5^ School of Materials Science and Engineering Nanyang Technological University 50 Nanyang Avenue Singapore 639798 Singapore

**Keywords:** asymmetric structure, droplet impact, horizontal laplace force, ice nucleation, superhydrophobic

## Abstract

Asymmetric structures have exhibited significant advantages in regulating wetting behavior. Nevertheless, the influence of this unique structural feature on anti‐icing performance remains to be further explored. In this work, static/dynamic anti‐icing performance is investigated on the asymmetric superhydrophobic structures fabricated by micro‐milling combined with electrodeposition. Notably, although the reduction of the degree of asymmetry increases the droplet adhesion force by augmenting the solid‐liquid interface, asymmetric structures can still enable the droplet to bounce off the surface through the horizontal Laplace force generated by the contact angle difference between the two sides of the droplet. On this basis, a dynamic behavior criterion for the droplet to detach from the surface is established at low temperatures. Molecular dynamics simulation indicates that the asymmetric structure can reduce the icing probability on the precursor film by inhibiting the nucleation and growth process of water molecules, decreasing the liquid‐ice interface, and reducing the adhesion under low temperatures. Generally, specific asymmetric structures with nucleation inhibition characteristics can reduce droplet adhesion and increase the driving force during the droplet retraction stage by enhancing the horizontal Laplace force, effectively improving the dynamic non‐wetting performance of the surface at even −40 °C.

## Graphical abstract

Special asymmetric structures with interfacial thermal resistance characteristics can not only provide time for droplet ejection by inhibiting the nucleation process inside the microstructure but also synchronously reduce droplet adhesion and increase the driving force during the droplet retraction stage by enhancing the horizontal Laplace force, significantly enhancing the dynamic non‐wetting performance of the surface at low temperatures.

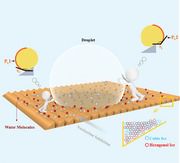

## 1 Introduction

Ice formation has become an important issue plaguing aerospace, wind electricity, rail transportation, and other fields due to its potential safety hazards and economic losses.^[^ ^1^, ^2^, ^3^, ^4^, ^5^, ^6^ ^]^ Considering the energy limitation and environmental friendliness of existing anti/de‐icing technologies, superhydrophobic materials have evolved as one of the ideal anti/de‐icing materials without energy consumption owing to their unique characteristics of low droplet adhesion, low ice nucleation rate, and low ice adhesion strength.^[^ ^7^, ^8^, ^9^, ^10^ ^]^ It is well known that the realization of anti/de‐icing function mainly depends on the coordinated control of structure and energy on a superhydrophobic surface.^[^ ^11^, ^12^, ^13^ ^]^ Notably, previous studies have shown that microstructure usually has a more significant impact on the hydrophobicity and anti‐icing performance of materials.^[^ ^14^, ^15^ ^]^ Therefore, numerous research works have been carried out to investigate the influence of microstructure on the anti/de‐icing property.

However, in consideration of the limitations of structural design and fabrication technology, conventional research on superhydrophobic structures is still focused on isotropic configurations, such as symmetric or irregular structures.^[^ ^16^, ^17^, ^18^ ^]^ Inspired by the structure of Trifolium, a periodic micro‐pit structure with higher critical Laplace pressure was constructed in order to achieve the reduction in ice accumulation by delaying the icing time.^[^ ^19^ ^]^ On this basis, an open micro‐cone structure with nanoparticles was designed to introduce two Cassie–Baxter states (CB I and CB II) into the surface by controlling the pinning behavior of the liquid during the cooling process, significantly enhancing the energy barrier between CB Wenzel states.^[^ ^20^ ^]^ Moreover, the influence of the wetting fraction of infiltrated liquid on the nucleation behavior was explored in the presence of potential nucleation sites on the regular micro‐column structure, promoting the establishment of design criteria for anti‐icing structure.^[^ ^21^ ^]^

Although the anti‐icing behavior of symmetric structures has been discussed from multiple scales and perspectives, the recent design concept of symmetric anti‐icing structures still focuses on prolonging the icing time.^[^ ^22^, ^23^, ^24^ ^]^ Interestingly, the asymmetric structure can significantly affect the mechanical behavior of droplets on superhydrophobic surfaces, which is expected to reduce ice accumulation on the surface by changing the form of solid‐liquid contact.^[^ ^25^, ^26^, ^27^, ^28^ ^]^ It was clarified that a superhydrophobic surface composed of asymmetric triangular microstructures with a lower droplet adhesion possessed the ability to make tiny droplets spontaneously bounce off the surface in a specific direction due to the anisotropic adhesion characteristics. This was the realization of directional long‐distance transport of droplets for the first time.^[^ ^29^ ^]^ Afterward, an asymmetric conical micro‐structure raised a “tip‐induced flipping” effect through the control of structure curvature and height gradient, which could further improve the droplet transport efficiency even in the condition of adverse Laplace pressure.^[^ ^30^ ^]^ Inspired by the structure of araucaria, an asymmetric 3D curved structure was designed to realize droplet movement in the direction of enlargement system energy without external energy input, breaking the inherent recognition that the direction of fluid movement was mainly determined by the solid surface structure.^[^ ^31^ ^]^ Subsequently, a tilted stepped mushroom‐like micropillar surface was introduced to reduce the contact time (7 ms) of low surface tension droplets (γ = 32 mN m^−1^) while controlling the droplet bouncing direction.^[^ ^32^ ^]^ On this basis, specific asymmetric inclined fiber structures could effectively accelerate the motion of the water droplet under the wind field, showing application potential in dynamic anti/de‐icing fields.^[^ ^33^ ^]^ It is worth noting that the current research about the influence of asymmetric structure on surface properties is still restricted to the directional motion control of droplets. Although researchers have tried to design spring‐like pillars, which can achieve the self‐ejection of droplets after freezing by relying on the releasement of elastic energy.^[^ ^34^ ^]^ Insufficient attention and exploration have been paid to its effect on anti‐deicing performance, especially nucleation behavior and static/dynamic non‐wettability at low temperatures.

Herein, static/dynamic anti‐icing performance was investigated on asymmetric superhydrophobic structures fabricated by micro‐milling combined with the electrodeposition method. Although the reduction of the degree of asymmetry increased the droplet adhesion force droplets by augmenting the solid‐liquid interface, asymmetric structures could still enable the droplet to bounce off the surface through the horizontal Laplace force generated by the contact angle difference between the two sides of the droplet. Molecular dynamics simulation indicates that the asymmetric structure could reduce the icing probability on the precursor film by inhibiting the nucleation and growth process of water molecules, decreasing the liquid‐ice interface, and reducing the adhesion force of droplets at low temperatures. This work confirms that specific asymmetric structures could simultaneously reduce droplet adhesion and increase the driving force during the droplet retraction stage by enhancing the horizontal Laplace force, providing theoretical guidance for reducing the probability of icing by controlling the contact time of droplets.

## 2 Results and Discussion

### 2.1 Fabrication and Characterization of Asymmetric Micro‐Nanostructures

**Figure 1:**
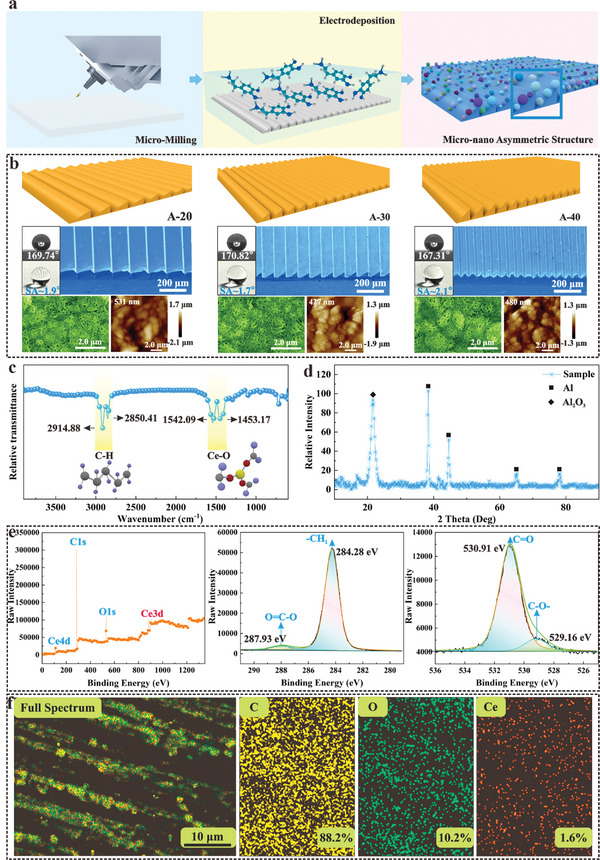
Fabrication and characterization of asymmetric micro‐nanostructures. a) Schematic diagram of the preparation process for hierarchical structure. b) The morphology of asymmetric micro‐nanostructures observed by SEM and AFM and the water contact angles on different surfaces are inserted into the corresponding SEM images. c) Surface chemical composition analysis by FTIR. d) GIXRD analysis of asymmetric micro‐nanostructure surfaces. e) XPS analysis of asymmetric micro‐nanostructures. The details of peaks O1 and C1 are indicated respectively. f) EDS analysis of asymmetric micro‐nanostructures.

An asymmetric array microstructure distributed on aluminum alloy was manufactured using micro‐milling technology. Subsequently, superhydrophobic treatment was performed on the asymmetric array microstructures by a one‐step electrodeposition method with cerous nitrate and stearic acid, as shown in **Figure** **1**a. Considering the comparability and manufacturability of the microstructure, the height of the microstructure was fixed and the degree of asymmetry was controlled by changing the structural angle, therein, the asymmetry degree is defined as the ratio of two sides of a microstructure. Three typical wedge‐shaped microstructures with an identical height of 50 µm and different angles are selected to investigate the effect of geometric parameters of asymmetric structures on non‐wetting performance under low temperatures (A‐20 sample with an angle of 20° and an asymmetry degree of 2.92, A‐30 sample with an angle of 30°and an asymmetry degree of 2 and A‐40 sample with an angle of 40°and an asymmetry degree of 1.56), as illustrated in Figure 1b. The contact angles of A‐20, A‐30, and A‐40 samples with superhydrophobic treatment are 169.74°, 170.82°, and 167.31°, respectively, while the sliding angles remain ≈2°.

Scanning Electron Microscope (SEM) images reveal that the superhydrophobic surfaces are composed of staggered nanorods with submicron length and a width of ≈50 nm. Meanwhile, Atomic Force Microscope (AFM) results indicate that the root‐mean‐square roughness (Rqs) of these three samples are 531, 527, and 480 nm, respectively. This demonstrates that the superhydrophobic nanostructures superimposed on the asymmetric microstructures have little effect on the characteristic morphology of the hierarchical structures. Additionally, Fourier Transform Infrared Spectrometer (FTIR), Grazing Incidence X‐Ray Diffraction (GIXRD), and X‐Ray Photoelectron Spectroscopy (XPS) methods were employed to further confirm the composition of the superhydrophobic surface, as depicted in Figure 1c–e. The absorption peaks occurring at 2914.88 and 2850.41 cm^−1^ are recognized as the ─CH_2_ group, and the absorption peaks reflecting ≈1542.09 and 1453.17 cm^−1^ are identified as cerium stearate.^[^ ^35^ ^]^

According to GIXRD analysis, few intrinsically hydrophobic cerium compounds (such as CeO_2_) exist on superhydrophobic surfaces. Corresponding XPS results also confirm that the energy spectrum peak near 284.28 eV is supposed to be ─CH_2_, while the peak ≈287.93 eV is deemed to be an O═C─O─ bond. Moreover, the energy spectrum peaks appear at 530.91 eV and 529.16 eV are authenticated as C═O and C─O─ bonds, respectively.^[^ ^35^ ^]^ It is worth noting that Ce3d peaks were detected in the full spectrum (≈885.12 and 903.04 eV), verifying the existence of cerium in the material as Ce^3+^.^[^ ^36^ ^]^ Furthermore, the atomic occupancy ratios of C, O, and Ce elements on the superhydrophobic surface are 88.2, 10.2, and 1.6%, respectively, with a relative ratio of 55.13:6.38:1 (similar to the 54:6:1 of [CH_3_(CH_2_)_16_COO]_3_Ce), indicating that the main component of the superhydrophobic surface is cerium stearate, as shown in Figure 1f.

### 2.2 Static Anti‐Icing Performance Evaluation

**Figure 2:**
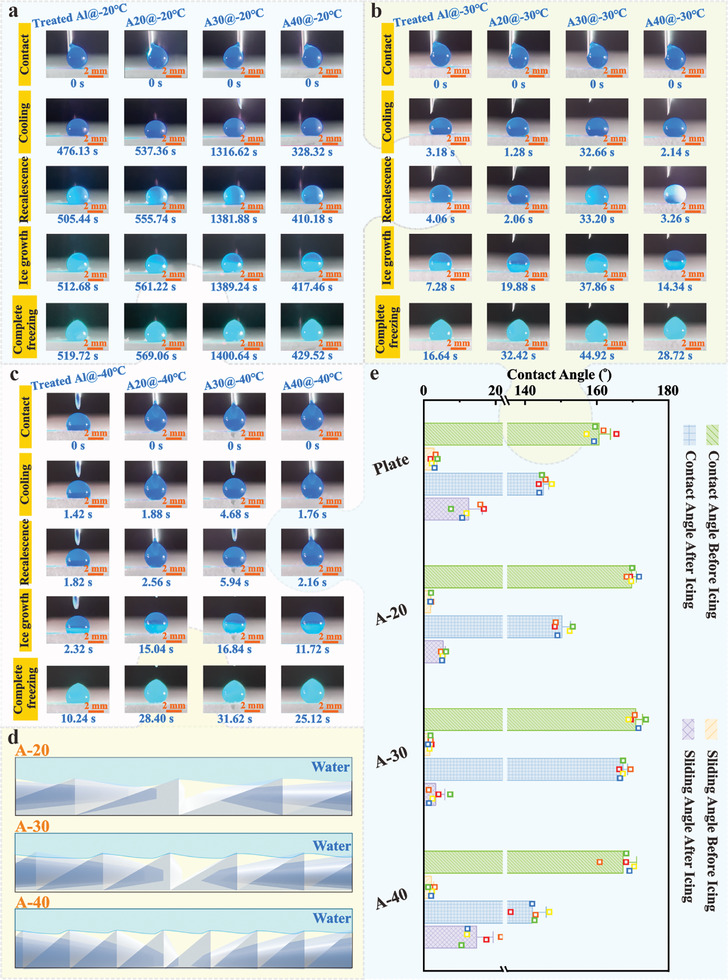
Icing delay behavior and ice‐melting process on different superhydrophobic surfaces. a) Icing delay process on various superhydrophobic surfaces at −20 °C. b) Icing delay process on various superhydrophobic surfaces at −30 °C. c) Icing delay process on various superhydrophobic surfaces at −40 °C. d) Schematic diagram of solid‐liquid contact behavior on different asymmetric micro‐nanostructures. e) Ice‐melting behavior on different superhydrophobic surfaces.

The same electrodeposition treatment was applied to an aluminum alloy plate as a comparison sample to evaluate the effect of asymmetric micro‐nanostructure on the icing delay characteristic. The droplet shows a recalescence phenomenon on the superhydrophobic plate after 505 s and completely froze within 14 s, as demonstrated in **Figure** **2**a. This indicates that the [CH_3_(CH_2_)_16_COO]_3_Ce deposited on the surface is sufficient to preserve a large amount of air at a low temperature of −20 °C, effectively prolonging the process of droplets icing. However, the recalescence time of the A‐20 sample is increased to 537 s while the complete freezing time is synchronously expanded to 32 s. It can be inferred that the asymmetric micro‐nanostructure effectively improves the air‐capturing capability of the surface, reducing the heat transfer efficiency between the droplet and the surface. Notably, the A‐30 sample exhibits a superior anti‐icing property under low temperatures with a recalescence time of 1316 s and a completely frozen time of 84 s. In contrast, the recalescence time of the A‐40 sample is 328 s, which is only 29.7% of that of the A‐30 sample, and even lower than that of the superhydrophobic plate. It is deduced that low temperature induces extra water molecule nucleation at the interface since the A‐40 sample with a lower asymmetry degree has a more solid‐liquid interface (higher quantity of microstructure per length), weakening the icing delay effect of the surface.

When the environment temperature is reduced to −30 °C, the recalescence time of the superhydrophobic plate sharply decreases to 4 s, while the anti‐icing ability of these three surfaces with asymmetric micro‐nanostructures also declines to varying degrees, as shown in Figure 2b. Unfortunately, the recalescence time of A‐30 is also shortened to 33 s, which is due to the fact that lower temperature significantly increases the icing nucleation rate at the solid‐liquid interface, allowing the droplets to further wet the structure, resulting in a sudden reduction in anti‐icing ability. Meanwhile, the recalescence time of A‐20 and A‐40 samples is only maintained for 2–3 s, which is only 6–9% of that on A‐30 samples. It can be inferred that the descending temperature greatly reduces the air pressure retained inside the micro‐nanostructures, resulting in an impairment of anti‐icing ability for A‐20 and A‐40 samples.

Figure 2c reveals that the recalescence and completely frozen time of the superhydrophobic plate are curtailed to 1 s and 10 s at −40 °C, respectively. Meanwhile, A‐20 and A‐40 samples both show a recalescence phenomenon within 2 s while the A‐30 sample can only delay the icing time to 5 s, indicating that lower temperature completely destroys the support effect of the air bubble inside the asymmetric micro‐nanostructure on the upper droplets. Generally, considering the geometric characteristics of the asymmetric micro‐nanostructure on the superhydrophobic surface, the less the asymmetry degree, the smaller the space occupied by droplets within the microstructure, as shown in Figure 2d. The microstructure with a lower asymmetry degree can retain more micro‐air pockets to hinder temperature transfer, leading to an improvement of anti‐icing performance. This is why the anti‐icing performance of the A‐20 sample is lower than that of the A‐30 sample. However, for droplets with a fixed size, the larger the asymmetry degree, the lower the solid‐liquid contact interfaces within the same contact radius. The lesser asymmetry degree tends to easily cause a wide range of direct temperature transfer from the low‐temperature substrate, promoting the nucleation of droplets, and resulting in an abnormal decrease in the icing delay time on the A‐40 sample.

Subsequently, an icing‐melting test is performed to further evaluate the anti‐icing performance of asymmetric superhydrophobic microstructures (Figure 2e). The freezing process of the droplets during the test is shown in the Supporting Information. It is discernible that the contact angle and sliding angle of the A‐30 sample can still be maintained at 167.31° and 3.3° after melting, respectively. In contrast, the A‐20 sample barely maintains the superhydrophobicity after the icing‐melting process (contact angle≈150.31°, sliding angle≈5.4°), while the A‐40 sample completely lost the superhydrophobicity (contact angle≈142.05°, sliding angle≈14.7°). It is affirmed that a tension gradient at the surface always prompts tiny bubbles trapped in the ice to the melt zone in a test process due to the Marangoni effect. The larger the temperature gradient, the greater the Marangoni force, leading to an inverted conical interface between the melted and non‐melted regions. These tiny bubbles can impact downward rapidly to restore the surface from the Wenzel state to the Cassie–Baxter state when the Marangoni force is greater than the sum of buoyancy and water resistance acting on the bubble. The critical conditions for bubble motion can be expressed as:^[^ ^22^ ^]^

(1) $$\int_{0}^{2\pi }\frac{d\gamma }{dT}\Delta T\cdot r_{b\;\max}d\alpha -\frac{4\pi }{3}\rho gr_{b\;\;\max}^{3}-\frac{\pi }{2}C\cdot \rho v_{b}^{2}r_{b\;\max}^{2}=0$$

where *dγ/dT* = 0.1 mN mK^−1^, *ΔT* is the temperature difference between the surface and the top of the droplet, *ρ* is the density of water, g is the gravitational constant, *r_bmax_* is the maximum bubble radius that can move downward, C is the resistance of water (5.9 × 10^3^) and *ν_b_* impact velocity of the bubble.

It can be seen from this equation that the bubble impact velocity increases exponentially with the increase of the temperature gradient between the droplet and the surface. Afterward, the evolution law of interfacial thermal resistance is investigated by establishing a quasi‐static heat flux model in order to further explore the relationship between the Marangoni effect and the wetting behavior.

(2) $$R_{Cassie}=\frac{1}{\pi r_{d}^{2}\sin^{2}\theta }\cdot \left(\frac{\delta_{c}}{k_{c}f_{m}f_{n}}+\frac{\delta_{m}}{k_{m}f_{m}}+\frac{\delta_{n}}{k_{n}f_{m}f_{n}}\right)$$

where *r_d_* is the radius of a droplet in the Cassie–Baxter state, *θ* is the apparent contact angle. *k_m_*, *k_c_*, *δ_m,_* and *δ_c_* represent the thermal conductivity and thickness of the substrate and superhydrophobic surface, respectively. *f_n_*, *k_n,_* and *δ_n_* are the area fraction, thermal conductivity, and microstructure height, respectively.

Hence, the superhydrophobicity can effectively ascend the interfacial thermal resistance, which not only effectively delays the icing process of the droplet, but also increases the temperature gradient between droplets and the surface, giving rise to an augment of bubble impact velocity. Synchronously, the A‐30 sample with higher icing‐delay performance also allows the ambient air to dissolve into the droplets sufficiently, providing enough tiny bubbles during the subsequent melting process.

### 2.3 Dynamic Non‐Wetting Performance at Low‐Temperature

**Figure 3:**
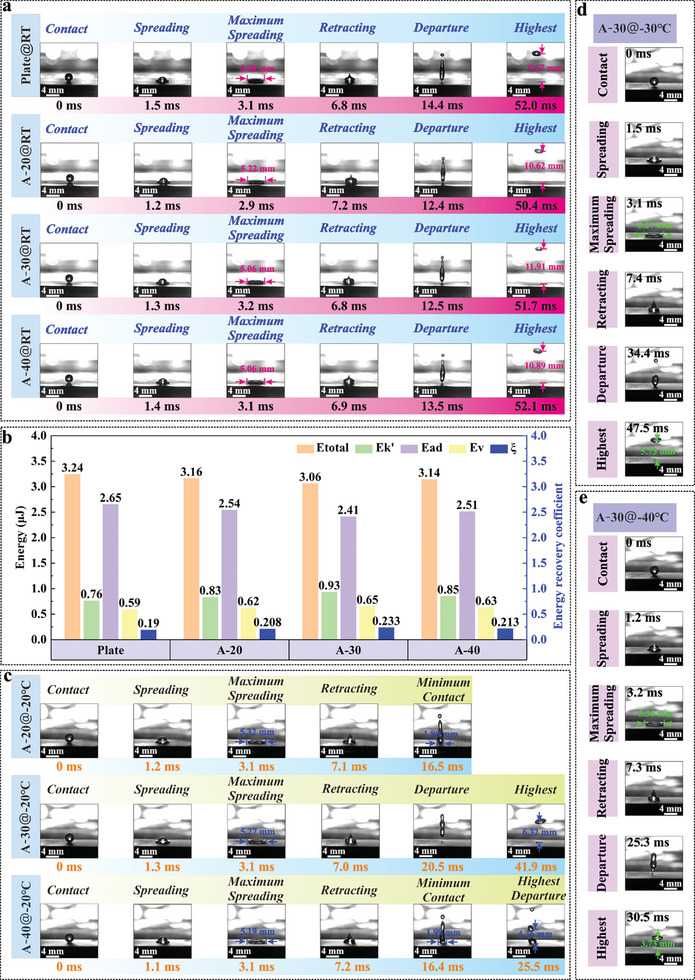
Dynamic non‐wetting behavior of various superhydrophobic surfaces. a) Droplet impact process on different superhydrophobic surfaces at room temperature. b) Energy dissipation during droplet impact process on various superhydrophobic surfaces. c) Droplet impact process on asymmetric micro‐nanostructures at −20 °C. d) Droplet impact process on A‐30 sample at −30 °C. e) Droplet impact process on A‐30 sample at −40 °C.

The bouncing processes of droplets on these three typical samples are monitored in order to investigate the dynamic non‐wetting behavior of asymmetric micro‐nanostructure, and the superhydrophobic plate is adopted as a comparison, as shown in **Figure** **3**a. Previous research reveals that the retraction process is the main factor determining whether droplets can detach from the superhydrophobic surface.^[^ ^37^ ^]^ Droplets can successfully bounce off the superhydrophobic plate within 11.3 ms after reaching the maximum spreading diameter. For the A‐20 sample with an asymmetry degree of 2.92, the retraction stage only takes 9.5 ms, which is 16% less than the retraction time on the superhydrophobic plate. Therefore, it is considered that the asymmetric micro‐nanostructure effectively decreases surface adhesion dissipation, shortening the contact time of droplets on the surface. Notably, the retraction stage of a droplet on the A‐30 sample is only 9.3 ms, even lower than that of the A‐20 sample. This indicates that the influence of the structure on the droplet adhesion behavior is directional due to its asymmetric characteristics. Structures with specific asymmetry exhibit the characteristic of inducing droplet contraction. Additionally, although the retraction time on the A‐40 sample increases to 10.4 ms, the contractive effect induced by asymmetric micro‐nanostructure still ensures that the contact time of the droplet on the A‐40 surface is shorter than that on the superhydrophobic plate.

Therewith, a equation is used to describe the energy conversion process in order to further explore the dynamic behavior of droplet impact:^[^ ^38^ ^]^

(3) $$E_{k}+E_{d}=E_{adhension}+E_{V}+{E^{\prime }}_{k}+{E^{\prime }}_{d}$$

where *E_k_* and *E_k_’* are the kinetic energy before and after the droplet contacts the superhydrophobic surface respectively, the *E_d_* and *E_d_’* are the surface energy before and after the droplet contact the surface. The *E_adhension_* is adhesive dissipative energy, and the *E_V_* is viscous dissipative energy.

Therein, the *E_k_* can be expressed as:

(4) $$E_{k}=\frac{2}{3}\pi \rho R_{0}^{3}V_{0}^{3}$$

where

(5) $$E_{v}=\mu {\left(\frac{V_{0}}{\delta }\right)}^{2}\tau \Omega$$

(6) $$\delta =4\frac{R_{0}}{\sqrt{2R_{e}}}$$

(7) $$R_{e}=\frac{\rho V_{0}R_{0}}{\mu }$$

(8) $$\Omega =\pi \delta R_{\max}^{2}$$

The *V_0_* is the instantaneous velocity of the droplet upon contact with the surface, taken as 1 m s^−1^, *R_0_* is the initial radius of the droplet, set as 1.22 mm, ρ is the droplet density of 1.0 × 10^3^ kg m^−3^, µ is the viscosity coefficient of water at room temperature, 1.0087 × 10^−3^ Nsm^−2^, and *R_max_* is the maximum spreading radius of the droplet during impact process.

On the premise of ignoring the air resistance and volume change before and after impact, the kinetic energy of the droplet after impact can be equivalent to the gravitational potential energy when it bounces to the highest point:

(9) $${E^{\prime }}_{k}=\left(\frac{4}{3}\right)\rho \pi R_{0}^{3}gH_{\max}$$

Considering the surface energy of the droplet is constant, the total energy consumption (*E_total energy loss_*) of the droplet during the impact process can be denoted as:

(10)

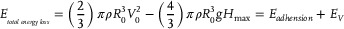




And the energy recovery coefficient *ε*, which can intuitively reflect the degree of surface energy dissipation can be calculated as:

(11) $$\varepsilon =\frac{{E^{\prime }}_{k}}{E_{k}}=\left(\frac{4}{3}\right)\rho \pi R_{0}^{3}gH_{\max}/\left(\frac{2}{3}\right)\pi \rho R_{0}^{3}V_{0}^{2}=\frac{2gH_{\max}}{V_{0}^{2}}$$

The calculation results indicate that the energy dissipation of droplets on all these samples is still dominated by adhesive dissipation. Particularly, the adhesion dissipation energy of droplets on the A‐30 sample is only 2.41 µJ, verifying the decisive role of droplet contraction characteristics induced by asymmetry structure on the bounce behavior during droplet impact, as illustrated in Figure 3b. Meanwhile, the viscous dissipation energy is always between 18.2% and 21.2% of the total dissipation energy during various impact processes, indicating that the internal flow inside the droplet has a slight influence on the bouncing process. Moreover, the energy recovery coefficients of different samples also confirm that asymmetric micro‐nanostructures can effectively reduce the contact process between droplet and surface, descending the adhesive dissipation energy.

When the surface temperature drops below zero (−20 °C), the droplet on the A‐20 sample is unable to detach from the surface with a retraction time of 13.4 ms, as delineated in Figure 3c. Relevant researches suggest that an extremely thin precursor film is presented at the leading edge of the solid‐liquid contact line at low temperatures, and the practical wetting process is the movement of the apparent contact line on the precursor film.^[^ ^39^, ^40^, ^41^ ^]^ Hence, the reduction of driving force for droplets bouncing off the surface may be due to the freezing of precursor film induced by low temperature, which changes the liquid‐solid interface into a liquid‐ice interface, resulting in an increasing adhesion dissipation. Unlike A‐20, although the A‐30 sample has a longer retraction time of 17.4 ms, the droplet can still detach from the surface. It is inferred that the droplet contraction induced by certain asymmetric structures provides an additional driving force and compensates for the adhesive dissipation caused by low temperature. Unfortunately, the droplet on the A‐40 sample could not escape from the surface in spite of the retraction time is only 13.3 ms. It can be considered that the bouncing behavior of droplets under low temperatures is determined by the competitive relationship between the contraction force induced by asymmetrical structure and the freezing rate of precursor film induced by low temperature. That is, the droplet can successfully detach from the low‐temperature surface when the contraction effect raised by the asymmetrical structure is greater than the liquid‐ice adhesion effect caused by precursor film freezing.

Subsequently, the A‐30 sample with excellent dynamic non‐wettability under low temperatures is further investigated at −30 °C. The results clarify that the decrease in temperature ascends the energy dissipation during the retraction stage, resulting in a reduction in bouncing height from 6.32 to 5.73 mm with a retraction time of 44.4 ms, as shown in Figure 3d. Surprisingly, the droplet can still detach from the A‐30 sample at −40 °C. Although the bouncing height has been reduced to 3.73 mm, its droplet retraction time has been actually shortened to 27.3 ms. This may be due to the enlargement of viscosity and tension of water molecules at the liquid‐ice interface triggered by low temperatures, promoting the contraction behavior of droplets. However, the augment of viscosity and tension is still hard to compensate for the adhesion dissipation caused by the precursor film icing occurring at larger areas, leading to a weakening of dynamic non‐wettability under low temperatures. This conclusion is also verified by the bouncing test of A‐20 and A‐40 samples in the Supporting Information (Figures S1 and S2, Supporting Information).

Further analysis of energy dissipation for the A‐30 sample at various low temperatures reveals that the continuous decrease of temperature elevates the adhesion dissipation energy from 2.41 to 2.97 µJ, confirming that adhesion dissipation is still the main type of surface energy dissipation at low temperatures, as shown in **Table** **1**. Furthermore, the viscous dissipation is basically maintained at ≈0.7 µJ below −10 °C (Figure S3, Supporting Information). This restates that the influence of temperature on the droplet movement for the A‐30 sample is realized through regulating the contraction behavior induced by the asymmetric structure and the liquid‐ice adhesion induced by the precursor film icing at the interface instead of controlling the viscosity and tension induced by interfacial heat transfer variation.

**Table 1:** Energy dissipation of A‐30 sample surface at different temperatures.

Temperature	*E_total_* [µJ]	*E_k_’* [µJ]	*E_adhesion_* [µJ]	*E_V_* [µJ]	*ε* [µJ]
Room Temperature	3.06	0.93	2.41	0.65	0.23
−10 °C	3.48	0.51	2.75	0.73	0.13
−20 °C	3.50	0.49	2.80	0.70	0.12
−30 °C	3.54	0.45	2.84	0.70	0.11
−40 °C	3.70	0.29	2.97	0.73	0.07

### 2.4 Mechanical Behavior of Droplet on Asymmetric Structures

**Figure 4:**
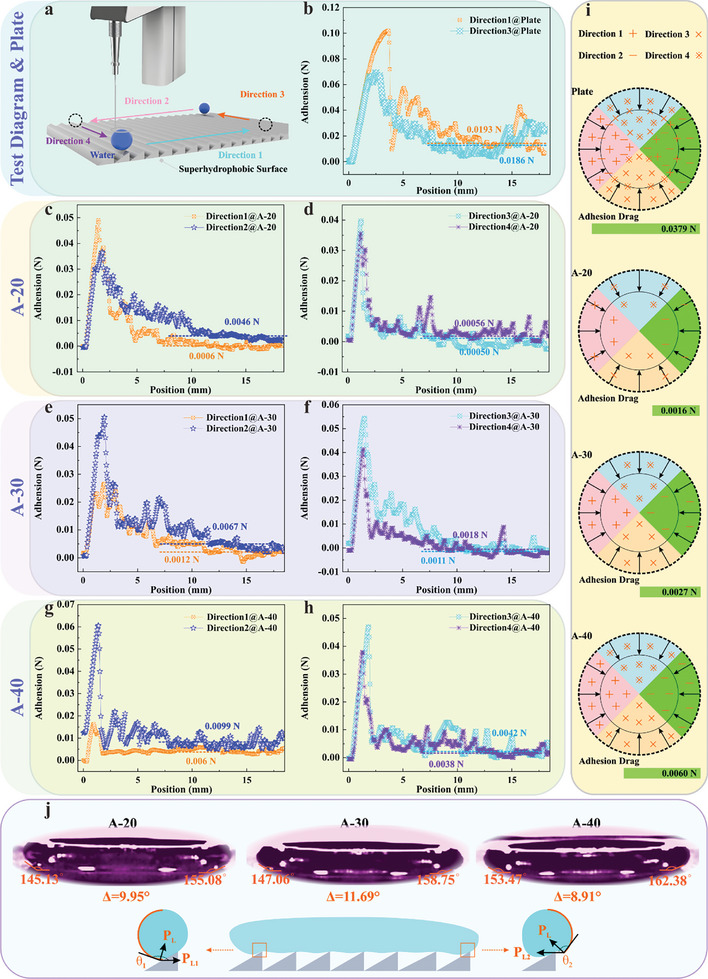
Analysis of droplet mechanical behavior on asymmetric structures. a) Diagram of droplet adhesion test. b) Adhesion strength of droplets on superhydrophobic plate along directions 1 and 3. c) Adhesion strength of droplets on A‐20 along directions 1 and 2. d) Adhesion strength of droplets on A‐20 along directions 3 and 4. e) Adhesion strength of droplets on A‐30 along directions 1 and 2. f) Adhesion strength of droplets on A‐30 along directions 3 and 4. g) Adhesion strength of droplets on A‐40 along directions 1 and 2. h) Adhesion strength of droplets on A‐40 along directions 3 and 4. j) Force analysis of droplet on asymmetric structures. i) Diagram of droplet adhesion behavior on different superhydrophobic surfaces.

Considering the remarkable effect of adhesive dissipation on droplet dynamic non‐wetting process, the contact behavior of droplets on the asymmetric structure is further investigated by monitoring the adhesive force of droplets moving along the asymmetric structure. Therefore, the hydrophobicity and adhesion properties are isotropic due to the uniform micro‐nanostructure on the superhydrophobic plate. Only two directions perpendicular to each other are selected for adhesive force evaluation. Moreover, the movement direction of the droplet facing the slop of the asymmetric microstructure is denoted as direction 1, while the opposite direction is defined as direction 2. Meanwhile, the directions along the ridge line of the asymmetric microstructure are denoted as directions 3 and 4, respectively, as shown in **Figure** **4**a.

The adhesion forces of droplets moving in two vertical directions on the superhydrophobic plate are 0.0186 N and 0.0193 N. It substantiates that the superhydrophobic micro‐nanostructures obtained by electrodeposition have isotropic wettability, as shown in Figure 4b. However, the adhesion force of the droplet moving along direction 1 on the A‐20 sample is 0.0006 N, which is only 13% of that in the opposite direction, as shown in Figure 4c. It is worth noting that the droplet adhesion forces are 0.0005 N and 0.00056 N in directions 3 and 4, respectively when droplets move along the ridge line of microstructures, which is similar to the droplet adhesion force in direction 1, as displayed in Figure 4d. Additionally, the adhesion force of the droplet moving toward direction 1 on the A‐30 sample is 0.0012 N, which is 17.9% of that in the opposite direction (Figure 4e). Interestingly, the adhesive force of droplets rolling along the ridge line is between 0.0011 N and 0.0018 N, which is slightly higher than that moving toward the slop side, as demonstrated in Figure 4f. Notably, the adhesion forces of droplets on the A‐30 sample are always higher than that on the A‐20 sample in various directions. This means that an A‐30 sample with a lower asymmetry degree brings a larger solid‐liquid contact area, increasing the adhesion effect between the droplet and the surface.

Moreover, the adhesive force is measured to be 0.006 N when the droplet moves toward direction 1 on the A‐40 sample, while the adhesive force of the droplet rolling in the opposite direction is 0.0099 N, as illustrated in Figure 4g. Simultaneously, the adhesive force of the droplets moving along the ridge line of the microstructure is only ≈0.004 N (Figure 4h). The higher adhesion force on A‐40 sample re‐confirms that the decrease in asymmetry degree leads to a significant enlargement in the solid‐liquid interface, which is also consistent with the conclusion obtained from Figure 2. Generally, the adhesive force of a droplet moving toward direction 1 is always smaller than that of a droplet rolling in the reverse direction on various asymmetry microstructures, demonstrating a tendency of the droplet to slide toward the slop of asymmetry microstructure. Nevertheless, the adhesion force of droplets shifting along the ridge line of the microstructure is similar. Considering the adhesion behavior of droplets on different surfaces, the force model of droplets is simplified, as shown in Figure 4i. The average droplet adhesion of the superhydrophobic plate is up to 0.0379 N, nevertheless, the adhesion force gradually increases with the decrease of asymmetry degree on asymmetric micro‐nanostructure surfaces (from 0.0016 N of A‐20 to 0.0060 N of A‐40).

Relevant research reveals that the droplet is also subjected to the horizontal Laplace force induced by the surface tension in addition to adhesion force during the impact process, which can be expressed as:^[^ ^42^ ^]^

(12) $$P_{Lx}=\frac{2\gamma \;\sin\theta_{i}}{R}$$

where *γ* is liquid surface tension, R is the radius of curvature for a droplet edge and *θ_i_* is the contact angle at the edge.

The lower the contact angle of the droplet, the greater the horizontal Laplace force. The contact angle at both sides of the droplet increases with the reduction of the asymmetry degree. Particularly, the deviation between contact angles does not vary linearly, where the contact angle difference on the A‐30 surface is higher by 11.69°, followed by the A‐20 sample with a deviation value of 9.95°, and the A‐40 sample indicates a lower difference value of 8.91°, as delineated in Figure 4j. Taking into account the distinction of horizontal Laplace force induced by contact angle deviation, the total horizontal Laplace force during the droplet retraction stage can be defined as:

(13) $$P_{L}=P_{L1}-P_{L2}=\frac{2\gamma \sin\theta_{1}}{R_{1}}-\frac{2\gamma \sin\theta_{2}}{R_{2}}$$

Therefore, the A‐30 surface with a larger contact angle difference generates a greater contraction force (which is the contraction driving force described in the previous section). Considering that the A‐30 sample, which can promote the droplet bounce off the surface at low temperatures, possesses a higher adhesion force than the A‐20 sample, it is confirmed that the horizontal Laplace force induced by asymmetric structure is the main factor determining whether the droplet can bounce off the surface.

Based on the above analysis and Equation 3, the energy variation process of a droplet from the maximum spreading stage to bouncing off the surface can be expressed as:

(14) $$E_{k}+E_{d}={E^{\prime }}_{k}+{E^{\prime }}_{d}+E_{adhension}+E_{V}-E_{p}$$

where *E_p_* is the energy dissipation driven by Laplace force.

Therein

(15) $$E_{d}=\frac{1}{4}\pi D_{\max}^{2}\sigma \left(1-\cos\theta \right)$$

(16) $${E^{\prime }}_{d}=\frac{4}{3}\pi R_{0}^{3}\sigma$$

(17) $$E_{adhesion}=\;\int \int \;\bar{f}Rd\alpha dR=\int_{0}^{2\pi }d\alpha \int_{0}^{R}\bar{f}RdR$$

(18) $$\begin{array}{ccc} E_{p} & = & \;\int \int \;\frac{2\gamma \sin\theta_{1}}{R_{1}}-\frac{2\gamma \sin\theta_{2}}{R_{2}}Rd\alpha dR\\ & = & \int_{0}^{2\pi }d\alpha \int_{0}^{R}\frac{2\gamma \sin\theta_{1}}{R_{1}}-\frac{2\gamma \sin\theta_{2}}{R_{2}}RdR \end{array}$$

where σ is the surface tension, *D_max_* is the maximum spreading diameter and the $\bar{f}$ is the average droplet adhesion on the cryogenic substrate.

Subsequently, the *E_k_’* needs to be larger than zero if the droplet can successfully bounce off the surface. Hence, a dynamic criterion for droplets to bounce off the surface at low temperatures is initially established:

(19) $$\begin{array}{ccc} & & \underset{0}{\int^{2\pi }}d\alpha \underset{0}{\int^{R}}\frac{2\gamma \sin\theta_{1}}{R_{1}}-\frac{2\gamma \sin\theta_{2}}{R_{2}}\mathrm{RdR}-\underset{0}{\int^{2\pi }}d\alpha \underset{0}{\int^{R}}\bar{f}\mathrm{RdR}\\ & & \;\geq \mu {\left(\frac{V_{0}}{\delta }\right)}^{2}\tau \Omega +\frac{4}{3}\pi R_{0}^{3}\sigma -\frac{1}{4}\pi D_{\max}^{2}\sigma \left(1-\cos\theta \right) \end{array}$$

### 2.5 Evolution of Ice Nucleation on Asymmetric Structures

Considering that the icing rate of precursor film on asymmetric micro‐nanostructures also plays an important role in droplet adhesion behavior under low temperatures, the icing process is evaluated by the nucleation rate in order to further analyze the effect of temperature on the icing behavior of precursor film on A‐30 sample with excellent dynamic non‐wettability:^[^ ^43^ ^]^

(20) $$J_{\phi }=\Phi K\left(T_{s}\right)e^{-\frac{\Delta G_{hete}}{k_{B}T_{s}}}$$

Ф is the contact fraction of the solid‐liquid interface and is defined as:

(21) $$\Phi =\frac{\cos\theta +1}{\cos\theta_{r}+1}$$

where *θ* is the apparent contact angle of sample A‐30 (taken as 170°), *θ_r_* is the intrinsic contact angle of [CH_3_(CH_2_)_16_COO]_3_Ce surface (set as 130°), *T_s_* is the substrate temperature, *ΔG_hete_* is critical nuclear barrier of non‐uniform nucleation at the solid‐liquid interface, k_B_ is a Boltzmann constant of 1.38 × 10^−23^ J K^−1^, and the K is the diffusion kinetic flux of water molecules at the ice‐water interface which can be expressed as:

(22) $$K\left(T_{s}\right)=\frac{k_{B}T_{s}n}{h}\cdot e^{-\frac{\Delta F_{diff}}{k_{B}T_{s}}}$$

Therein, n is the number density of water molecules at the ice‐water interface (3.34 × 10^28^), h is the Planck constant of 6.63 × 10^−34^ J s^−1^, *ΔF_diff_* is the diffusion activation energy of water molecules across the ice‐water interface:^[^ ^44^ ^]^

(23) $$\Delta F_{diff}=\frac{k_{B}ET^{2}}{{\left(T-T_{R}\right)}^{2}}$$

where *E* is defined as 892 K and *T_R_* is 118 K.^[^ ^45^ ^]^

The critical nuclear barrier (*ΔG*) of the ice core is a function of temperature and interface energy. According to classical nucleation theory, the relationship between the free energy barrier of non‐uniform nucleation (*ΔG_hete_*) and the homogeneous nuclear energy barrier (*ΔG_homo_*) is as follows:

(24) $$\Delta G_{hete}=\Delta G_{\mathrm{hom}o}\cdot f$$

(25) $$\Delta G_{\mathrm{hom}o}=\frac{16\pi \gamma_{IW}^{3}}{3{\left(\Delta G_{\nu }\right)}^{2}}$$

(26) $$\gamma_{IW}=28.0+0.25\left(T_{s}-273.15\right)$$

(27) $$\Delta G_{\nu }=\frac{T_{m}-T_{s}}{T_{m}}\Delta H_{\nu }$$

where *γ_IW_* is the interfacial tension of the ice‐water interface, *ΔG_V_* is the difference in volume free energy between the ice and water phases, *T_m_* is the melting temperature of ice at standard atmospheric pressure (273.15 K), *ΔH_V_* is the volumetric enthalpy during water melting process (2.87 × 10^8^ J m^−3^), and f can be calculated by:^[^ ^43^ ^]^

(28) $$\begin{array}{ccc} f & = & \frac{1}{2}+\frac{1}{2}\left(\frac{1-mx}{w}\right)+\frac{x^{3}}{2}\left[2-3\left(\frac{x-m}{w}\right)+{\left(\frac{x-m}{w}\right)}^{3}\right]\\ & & +\frac{3mx^{2}}{2}\left(\frac{x-m}{w}-1\right) \end{array}$$

(29) $$m=\cos\theta_{IW}$$

(30) $$x=\frac{R_{a}}{R_{c}}$$

(31) $$w=\sqrt{1+x^{2}-2xm}$$

(32) $$\cos\theta_{Iw}=\frac{\gamma_{1}\cos\theta_{1}-\gamma_{w}\cos\theta_{w}}{\gamma_{IW}}$$

(33) $$r_{c}=-\frac{2\gamma_{IW}}{\Delta G_{\nu }}$$

where *θ_IW_* is the contact angle of the surface with roughness *R_a_* in the supercooled water, which is basically equivalent to the intrinsic contact angle of the superhydrophobic surface and *R_c_* is the critical nucleation size.

The calculation results indicate that the ice nucleation rate at the solid‐liquid interface increases exponentially with the decrease in temperature, as shown in **Table** **2**. The ice nucleation rate is lower than 1.94 × 10^−13^ at temperatures above −20 °C, meaning that only several water molecules can nucleate at the interface. However, the ice nucleation rate rises sharply to 2.79 × 10^18^ as the temperature further drops below −30 °C, demonstrating a wide range of ice behavior exists in the precursor film. The higher icing rate significantly increases the energy dissipation during the droplet impacting process and greatly weakens the non‐wetting characteristics of the superhydrophobic surface at low temperatures.

**Table 2:** Ice nucleation rate of A‐30 samples at various temperatures.

Temperature [k]	γ_*IW*_	Δ*G_v_* [× 10^7^]	*R_c_* [× 10^−9^]	*f*	$\Delta G_{\mathrm{hom}o}$ [× 10^−19^]	Δ*F_diff_* [× 10^−20^]	$J_{\Phi }$
263.15	0.0255	1.05	4.86	0.8216	25.2	4.05	≈0
253.15	0.0230	2.10	2.19	0.8215	4.62	4.32	1.94 × 10^−13^
243.15	0.0205	3.15	1.30	0.8215	1.45	4.65	2.79 × 10^18^
233.15	0.0180	4.20	0.86	0.8214	0.55	5.05	7.69 × 10^26^

**Figure 5:**
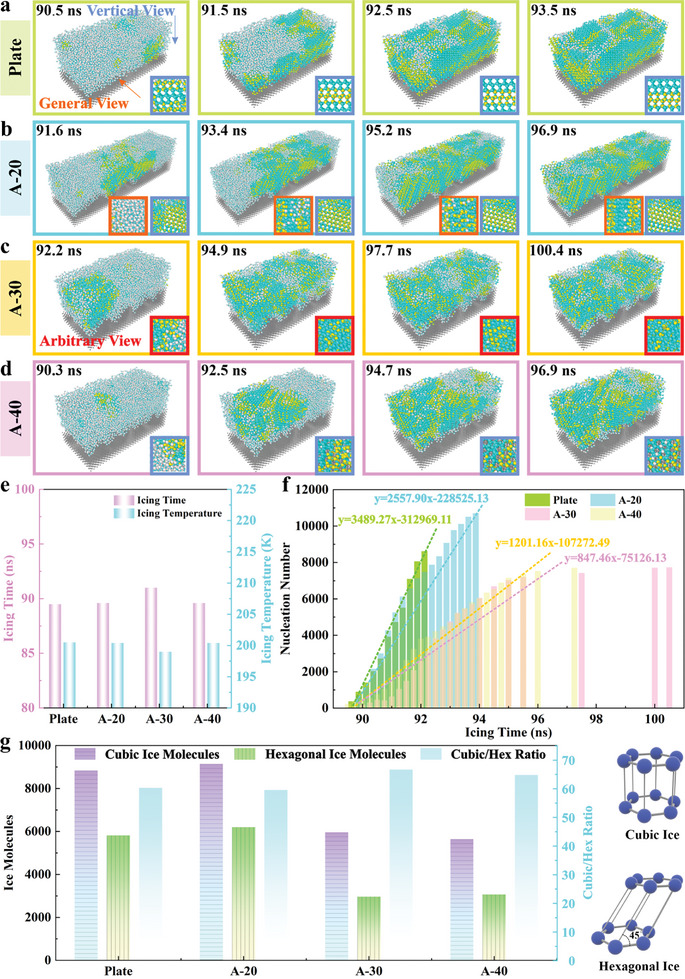
Evaluation of ice nucleation process on superhydrophobic surfaces. a) Molecular dynamics calculation of ice nucleation process on a superhydrophobic plate. b) Molecular dynamics calculation of ice nucleation process on A‐20 surface. c) Molecular dynamics calculation of ice nucleation process on A‐30 surface. d) Molecular dynamics calculation of ice nucleation process on A‐40 surface. e) Nucleation times and nucleation temperature for various superhydrophobic surfaces. f) Nucleation rate of various superhydrophobic surfaces. g) Statistics on the quantity of cubic ice and hexagonal ice on different superhydrophobic surfaces.

Subsequently, the icing process of water molecules on the superhydrophobic asymmetric structure is simulated by the molecular dynamics method in order to further explore the icing mechanism of the precursor film at the interface. The specific criterion for the nucleation process is provided in the Supporting Information. The simulation results show that nucleation sites appear at multiple locations above the superhydrophobic plate, and the mixed area of cubic ice (marked by blue molecules) and hexagonal ice (marked by yellow molecules) is formed. Afterward, several water molecules near the initial nucleation position are rearranged as the nucleation progresses, resulting in an abundant water molecule regularly arrayed in the vertical direction, as shown in the enlarged box (marked by blue) in **Figure** **5**a. Additionally, there are a certain number of water molecules regularly arranged in both the vertical direction and the general direction (illustrated in the enlarged box marked by orange) on the A‐20 surface (Figure 5b). Diversely, only a partial of arrayed water molecules can be discovered in the vertical direction on the A‐40 surface (Figure 5d). In particular, the A‐30 surface exhibits a frozen state with completely disordered, and few arrayed water molecules can be observed from any direction (Figure 5c). The corresponding nucleation temperature and nucleation time reveal that the nucleation time of the superhydrophobic plate is 89.5 ns, which is slightly lower than that of the A‐20 surface (89.6 ns), as depicted in Figure 5e and Figure S4 (Supporting Information). However, the nucleation time of 88.7 ns on the A‐40 surface is significantly shorter than that on a superhydrophobic plate, while the ice nucleation time on the A‐30 surface is up to 91 ns.

Considering that the icing process is essentially a regular arrangement of water molecules, ice tends to nucleate and grow rapidly along an easy growth direction, and the change of growth orientation is to adapt to the limitation of nucleation space. This change will disrupt the original growth inertia of ice, hindering the growth of the ice layer along the easy growth direction, and resulting in a delay in nucleation and growth. Compared with the previous simulation for symmetrical structure,^[^ ^46^ ^]^ the specific asymmetric structure can effectively induce the disordered arrangement of vast water molecules, promoting the continuous adjustment of ice crystal orientation during nucleation, significantly delaying the nucleation process and demonstrating the ability to suppress ice formation (Figure S5, Supporting Information).

The nucleation molecules at different icing times are calculated, as demonstrated in Figure 5f. The superhydrophobic plate exhibits a greater ice growth rate, which is 1.36 times higher than that on the A‐20 surface. Notably, the growth rate on the A‐40 sample is only 34.4% of that on the flat plate, indicating a certain superiority in inhibiting the icing process. Surprisingly, the A‐30 surface presents a lower growth rate, only 24.3% of that on the superhydrophobic plate. On this basis, further analysis of ice crystal type reveals that the cubicity (the proportion of cubic ice in all molecules) of ice molecules on the superhydrophobic plate is 60.3%, which is slightly higher than that of 59.57% on the A‐20 surface, as shown in Figure 5g. Meanwhile, the A‐30 surface has a superior cubicity of 66.72%, which is 1.93% larger than that of the A‐40 surface. As is well‐known, ice formation is the process in which water molecules fill the space without creating vacancies as much as possible. The limited growth space is mismatched with the ice type due to the unique spatial structure and growth orientation of cubic ice and hexagonal ice.^[^ ^47^ ^]^ Hexagonal ice exhibits a preferred growth tendency due to its anisotropic structure, and its nucleation energy barrier is much lower than that of cubic ice. Therefore, it is affirmed that the specific asymmetric structure (A‐30) can effectively delay the nucleation process while inhibiting the growth of ice crystals by simultaneously perturbing the directional arrangement of water molecules and increasing the content of cubic ice. This is why the nucleation probability of droplets on the A‐30 surface is lower at low temperatures during the impact process.

Generally, asymmetric structures can reduce the probability of ice formation in surface precursor films by suppressing the nucleation process of water molecules. Whereafter, less area of the precursor film is frozen due to a lower icing probability, leading to fewer liquid ice interfaces and a lower adhesion. It is considered that the ice suppression characteristics of asymmetric structure not only reduce the adhesion force at the solid‐liquid interface but also provide more time for droplets to escape from the surface. Particularly, the specific asymmetric structures (A‐30) can simultaneously reduce droplet adhesion and increase the driving force during the droplet retraction stage by enhancing the horizontal Laplace force. This also confirms that the asymmetry of the structure can promote the self‐ejection effect of the droplet at the multi‐scale level, effectively improving the dynamic non‐wetting performance of the surface under low temperatures.

## 3 Conclusion

In this work, asymmetric hierarchical structures with super‐hydrophobicity were fabricated by micro‐milling combined with the electrodeposition method. The static anti‐icing performance indicates that the greater the degree of asymmetry of the hierarchical structure, the more solid‐ice interfaces were generated below the droplet, and the extra thermal pathways were constructed for heat transfer under low temperatures, improving the ice crystal growth rate inside the droplet. Meanwhile, the asymmetric structure (A‐30) with higher icing delay performance could not only ascend the temperature gradient between the droplet and the substrate by increasing the interface thermal resistance but also provide more time for the air to fully dissolve into the droplet, improving the impact speed of the air bubble during the melting process, effectively promoting the surface wetting state to revert to the Cassie model.

The subsequent dynamic non‐wettability evaluation revealed that the energy dissipation of droplets on various superhydrophobic surfaces mainly manifested as adhesive dissipation, and the bounce behavior of droplets was dominated by the competitive relationship between the contraction driving force induced by asymmetric structure and the freezing rate of precursor film induced by low temperature. Further force analysis clarified that the decrease in asymmetry degree led to a significant enlargement in the solid‐liquid interface, increasing the adhesion force of droplets on a superhydrophobic surface. However, the difference in contact angles between the two sides of a droplet induced by asymmetric structure generated a horizontal Laplace force, which determined whether the droplet could bounce off the surface. On this basis, a dynamic behavior criterion for the droplet to detach from the surface was established at low temperatures. Furthermore, combined with molecular dynamics analysis, it was verified that asymmetric structure could reduce the icing probability in the precursor film by inhibiting the nucleation and growth process of water molecules, decreasing the liquid‐ice interface, and reducing the adhesion force of droplets under low temperatures. Generally, specific asymmetric structures with nucleation inhibition characteristics could simultaneously reduce droplet adhesion and increase the driving force during the droplet retraction stage by enhancing the horizontal Laplace force, effectively improving the dynamic non‐wetting performance of the surface at even −40 °C.

## 4 Experimental Section

### Preparation of Superhydrophobic Asymmetric Structure

Before the electrodeposition process, the samples of 2024 aluminum alloy were polished, and then milled layer by layer through five‐axis precision micro‐milling equipment until the asymmetric micron structure was obtained. Afterward, the electrodeposition processes were performed on a direct current power with a constant voltage of 30 V and an electrodeposition of 10 min at 40 °C. The electrolyte system was stearic acid (C_18_H_36_O_2_, 0.0008 mol L^−1^) and cerium nitrate (Ce(NO_3_)_3_·6H_2_O, 0.0002 mol L^−1^). After electrodeposition, the samples were dried at 80 °C on the hotplate for 24 h. The detailed preparation process and the corresponding chemicals and reagents used in this work are clarified in the Supporting Information.

### Material Characterizations

The surface topography was observed by a field emission scanning electron microscope (FE‐SEM, Hitachi S4800, Japan) equipped with energy dispersive X‐ray spectroscopy (EDS) and an atomic force microscope (AFM, Bruker Dimension ICON). The chemical composition on the surface was analyzed by Fourier‐transformed infrared spectroscopy (FTIR, Nicolet IN10, ThermoFisher), X‐ray photoelectron spectroscopy (XPS, Thermo Scientific K‐Alpha), and Grazing Incidence X‐ray diffractometry (GIXRD, D8 ADVANCE, Bruker). Additionally, the static non‐wettability was measured by a contact angle analyzer (Kruss DSA100, Germany). The specific test process and parameter setting are listed in the Supporting Information.

### Static Anti‐Icing Performance Evaluation

The icing delay behavior and the melting process of a single water droplet ≈6 µL were observed by a Charge Coupled Device camera at a temperature regulation platform. The relative humidity of the test environment was kept within 5%. The asymmetric structure sample was placed on the platform at −20, −30 and −40 °C respectively, and the droplet was located in the center of the sample to observe the freezing process. Moreover, a small amount of sodium fluorescein was added into the droplet in order to distinguish the recalescence phenomenon clearly. Subsequently, the low‐temperature platform was heated to 25 °C at a rate of 5 °C min^−1^, and the contact angle and sliding angle of droplets on the surface were measured with a contact angle analyzer. The average values were achieved by at least five repetitive tests.

### Dynamic Non‐Wettability Analysis

The dynamic non‐wettability property was determined by a high‐speed camera (Photron Mini 100) with a frame of 10 000 s^−1^. The impact and ricochet processes were observed by a droplet ≈8 µL. Moreover, the droplet was released from a fixed height of 50 mm over the surface, and the initial impact velocity was ≈1 m s^−1^, following the equation $v=\sqrt{2gh}$. Meanwhile, an adhesion tester (German Kruss K100) was adopted to measure the droplet adhesion in order to investigate the motion characteristics of droplets on superhydrophobic surfaces. The droplet used in this test was ultra‐pure water with a volume of 20 µL. The droplet movement speed was recognized as 40 mm min^−1^, and the movement distance was set to 18 mm to ensure that the droplet always moved within the asymmetric microstructure region.

### Molecular Dynamics Simulation Method

The LAMMPS software was employed to simulate the icing process of water molecules on various surfaces. The model used in this simulation was scaled down in order to ensure the accuracy of the calculation under the premise of reducing the computing resources, as depicted in Figure S6 (Supporting Information). Therein, the mW coarse‐grained potential and the LJ potential were adopted to describe the interaction among water molecules and the force between water molecules and the matrix, respectively. Particularly, the interaction energy between water molecules and matrix was assigned to 0.12 kcal mol^−1^, which was consistent with the actual wetting state of the superhydrophobic surface in this work, as shown in Figure S7 (Supporting Information). The specific simulation parameters were demonstrated in the Supporting Information.

## Conflict of Interest

The authors declare no conflict of interest.

## Supporting information



Supporting Information

## Data Availability

The data that supports the findings of the study are available from the corresponding author.
