# Supplementary material for: Excellent Dynamic Non‐Wetting Performance Induced by Asymmetric Structure at Low Temperatures: Retraction Actuation and Nucleation Inhibition
Source: Adv Sci (Weinh). 2025 Feb 28;12(16):2500590. doi: 10.1002/advs.202500590 (PMC12021054; doi:10.1002/advs.202500590)
Supplement: Supplementary file 1 — Supporting Information [file ADVS-12-2500590-s001.docx]

Supporting Information

Excellent Dynamic Non-Wetting Performance Induced by Asymmetric Structure at Low Temperatures: Retraction Actuation and Nucleation Inhibition

Jiawei Jiang^1^, Yizhou Shen^1^*, Yangjiangshan Xu^2^, Zhen Wang^1^, Senyun Liu^3^, Yanyan Lin^4^, Jie Tao^1^, Zhong Chen^5^

1. Results and Discussion

1.1. Static Anti-icing Performance Evaluation

Usually, the icing-melting process of droplets on the asymmetric micro-nanostructure can be divided into three stages:^[1]^ (1) cooling and condensation stage, (2) Ice nucleation stage, (3) Freezing stage. The transformation of surface wetting morphology often occurs in the first stage, where the strong van der Waals forces inside the hierarchical structure cause condensation of surface water vapor, leading to an increase in droplet contact diameter. With the continuous decrease of temperature, the droplet starts to nucleate and the air bubble between the droplet and the structure is gradually pierced, resulting in a decrease in non-wetting performance. Meanwhile, the solubility of air in water increases due to the decrease in temperature, giving rise to a formation of tiny bubbles. On the contrary, the ice layer gradually melts from the bottom at the beginning of the melting stage, and the melting process at the bottom is difficult to synchronize. There is a tendency for the bottom of the droplets to spread outward. As the melting stage continues, a significant difference in surface tension between the melted and non-melted areas is appeared due to the temperature gradient inside the droplets. The higher the temperature, the lower the surface tension in the melted area, and the lower the temperature, the higher the surface tension in the non-melted area.

Based on a larger temperature gradient, abundant bubbles can move along the generatrix of the inverted conical phase interface at high speed until they detach from the vertex, promoting the recovery of air bubbles in superhydrophobic surface during the melting stage. With the continuous recovery of the air bubble, the interface thermal resistance also increases, causing an enlargement in the impact velocity of the bubbles until they stop moving due to liquid resistance and reach equilibrium. The massive downward impact of these bubbles causes the surface to gradually changes from a "Wenzel" state to a "Cassie" state during the melting process.

1.2. Dynamic Non-Wetting Performance at Low-Temperature

The droplet on the A-20 sample not only cannot bounce off the surface, but also the minimum contact diameter of the droplet is increased to 2.60 mm at -30 ℃ (~1.80 mm at -20 ℃), which means that the lower temperature also increases the adhesive dissipation and viscous dissipation simultaneously, reducing the driving force of droplet ejection, as shown in **Figure S1**. Similarly, the minimum contact diameter of the droplet is further enlarged to 2.71 mm at -40 ℃. This also indicates that the decrease of temperature improves the energy dissipation during the retracting process.


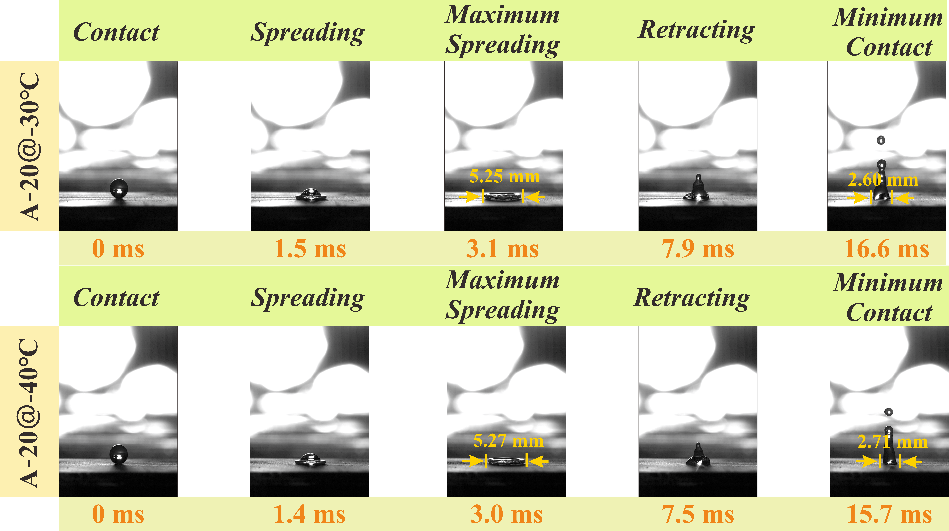


**Figure S1**. The impact process of droplets on A-20 surface at -30℃ and -40℃.

The A-40 sample with a lower asymmetry degree has a more solid-liquid interface, which promotes the icing process of the precursor film at -30 ℃, as depicted in **Figure S2**. Therefore, the adhesion effect between the droplets and the underlying ice layer is significantly enhanced, and the minimum contact diameter of the droplet is increased from 1.99 mm (-20 ℃) to 2.34 mm. With the further decrease in temperature, the mass of droplets that can eventually break off the A-40 surface also decreases significantly, as shown in **Figure S3**. Meanwhile, the rebounding height of fractured droplet is even only 3.46 mm, which is 1.21 mm lower than that at -30 ℃. This implies that an excessive increase of the solid-liquid interface can also promote the droplet viscosity and surface tension to dominate the dynamic non-wetting process at lower temperatures.


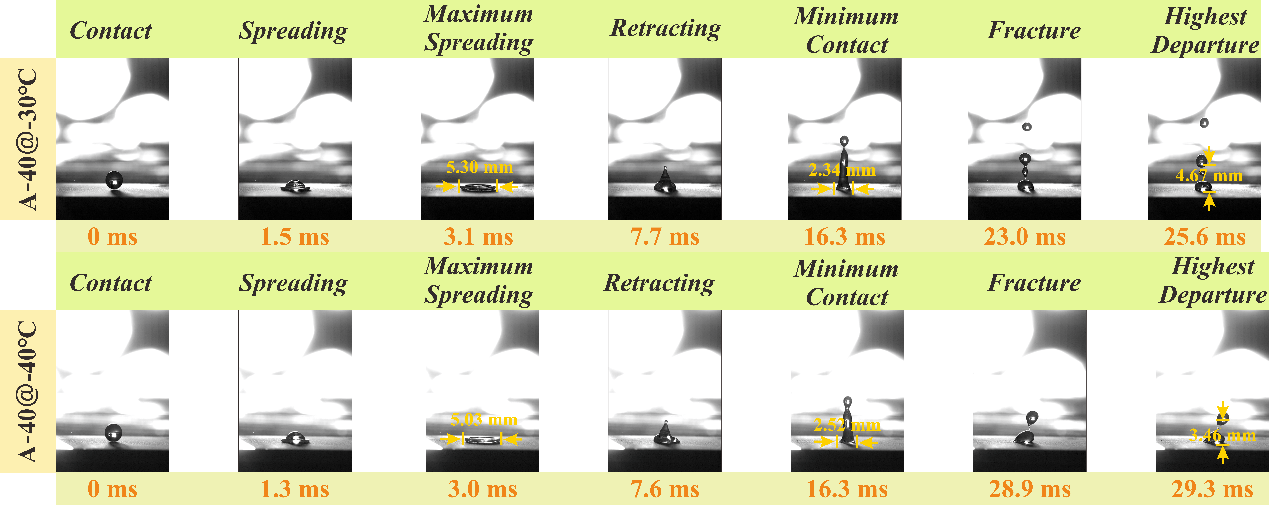


**Figure S2.** The impact process of droplets on A-40 surface at -30 ℃ and -40 ℃.


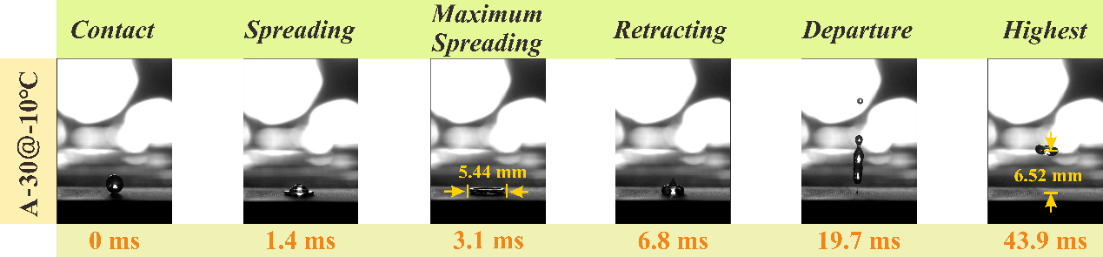


**Figure S3**. The impact process of droplets on A-30 surface at -10 ℃.

1.3. Evolution of ice nucleation on asymmetric structures

The ice nucleation is the ordering process of disordered water molecules. The icing process can be captured by monitoring the structure of water molecules.^[2]^ Water molecules in ice are usually arranged in a cubic or hexagonal structure, and the ice structure can be identified using a molecular visualization software OVITO. The recognition path of this software is as follows:^[3]^ First, the nearest neighbors of an atom are identified. Then, for each of these four neighbors, their respective nearest neighbors are identified. This yields the list of second nearest neighbors of the central atom. Finally, the CNA fingerprint is computed for these 12 second nearest neighbors and the central atom. If they are arranged on an FCC lattice. Then, the central atom is classified as a cubic diamond. If they form an HCP structure. then the central atom is marked as a hexagonal diamond atom.

The free energy of ice is always lower than that of water under supercooled conditions, hence, there has been a sudden drop in system energy when water molecules start to nucleate. The total potential energy data show that there are four stages in the freezing process: (1) a long quiescent period with relatively constant potential energy; (2) a short period during which the potential energy slowly decreases; (3) a short period during which the potential energy decreases rapidly; and (4) a final period with reduced but relatively constant potential energy and during which the ice structure fully forms. Water in the quiescent period is in a supercooled liquid state, exhibiting intermittent collective motions and energy fluctuations associated with hydrogen bond rearrangements. The freezing process starts in stage (2). The fact that the system explores the overall relatively flat potential energy landscape for a considerable time (that is, the quiescent period) before entering the fast-growing period agrees with the predictions of basic nucleation theory 3,4,5. However, the MD simulation also provides a molecular-level illustration of the water freezing process not obtainable from conventional nucleation theory 5. The corresponding nucleation temperature and time can be achieved from **Figure.S4**.


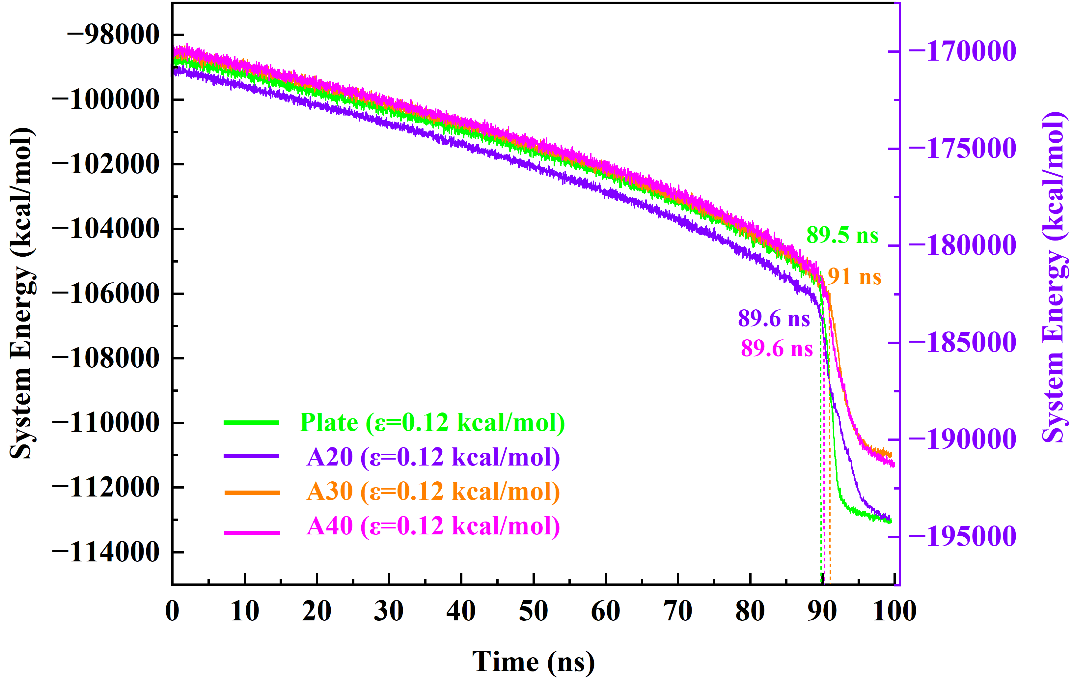


**Figure S4**. Energy curves of ice nucleation on different surfaces with ε=0.12 kcal mol^-1^.

As shown in **Figure S5**, nucleation of water molecules on the plate surface (the corresponding parameter settings are consistent with the references in **Figure. 5b**) occurs after 88.7 ns, as depicted in **Figure.5a**. For asymmetric structures, the A-20 surface has a longer freezing time of 84.6 ns, while the icing time of A-40 surface is slightly shorter of 79.9 ns, showing a weaker icing delay effect. Notably, the A-30 surface indicates a lower freezing time of only 69.8 ns, which greatly promotes the nucleation process of water molecules. However, the previous research reveals that symmetrical structures with a 40° angle exhibit a more pronounced tendency towards icing than structures with a 30° angle, as demonstrated in Figure.5b. This clarifies that the angle is not the only factor affecting the nucleation of water molecules on the structure surface, and the asymmetry degree also plays a significant role in icing delay process.


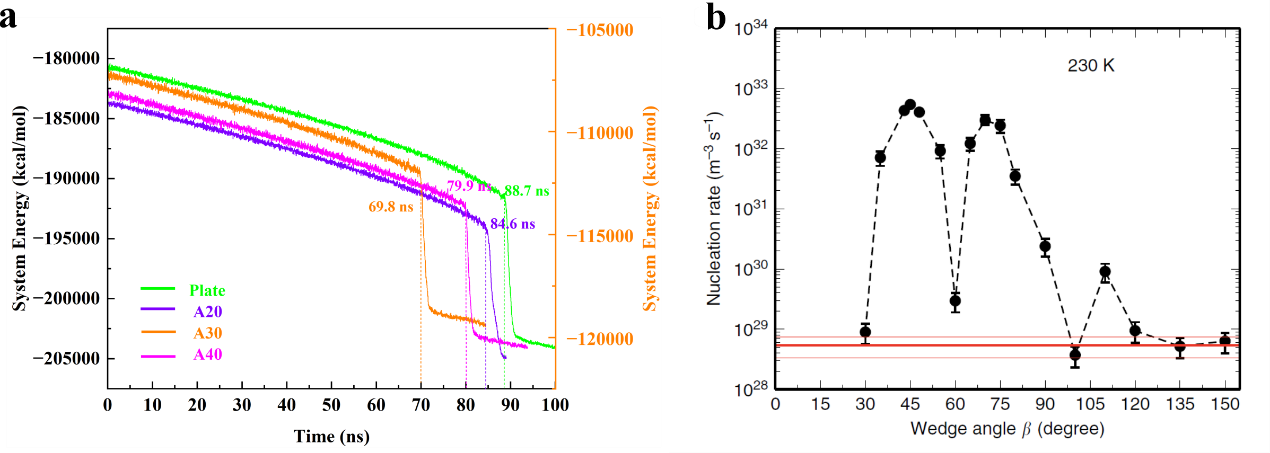


**Figure S5.** (a) ice simulation of asymmetry structures in this work, (b) ice simulation of symmetrical structures in previous work.^[4]^

2. Experimental Section/Methods

Preparation of Superhydrophobic Asymmetric Structure

Before the electrodeposition process, the specimens were polished with a series of emery papers (from 240# to 2000#), then mechanically polished to smooth, therewith, cleaned ultrasonically in acetone, ethanol and deionized water for 30 min, respectively. Afterwards, the sample was processed layer by layer by micro-milling with a precision of 0.2 microns to the corresponding morphology. Before the electrodeposition process, the milled sample was rapidly oxidized in an acid solution (NaF·CrO_3_·H_3_PO_4_·H_3_BO_3_) for 45 s to obtain the surface oxide layer in order to improve the surface uniformity during the subsequent electrodeposition processes. Therein, chromic anhydride (CrO_3_) of 0.04 mol L^-1^ is used to oxidize the aluminum on the surface. The introduction of phosphoric acid (H_3_PO_4_, 0.22 mol L^-1^) and boric acid (H_3_BO_3_, 0.13 mol L^-1^) improves the compactness of oxide film. Also, the formation rate of the oxide layer is improved by the addition of sodium fluoride (NaF, 0.12 mol L^-1^).

The electrodeposition processes were performed on a direct current power with a constant voltage of 30 V and an electrodeposition of 10 min at 40 ℃, where the specimen was used as the cathode and the platinum plate as the anode. The distance of the two electrodes was defined as 2 cm. Cerium nitrate hexahydrate (Ce(NO_3_)_3_·6H_2_O, 0.0002 mol L^-1^) and the stearic acid (C_18_H_36_O_2_, 0.0008 mol L^-1^) immersed in ethanol were used as the electrolyte solution. In addition, the volume of the electrolyte solution was set as 100 mL. After deposition, the sample was carefully removed from the electrolyte and rinsed thoroughly with ethanol, thereafter dried at 80 ℃ on the hotplate for 24 h.

The square plates of 2024 aluminum alloy (30 mm × 30mm) with a thickness of 2 mm were purchased from South West Aluminum Industry Group Co., Ltd. Cerium (III) nitrate hexahydrate and stearic acid were obtained from Shanghai Aladdin Biochemical Technology Co., Ltd. Ethanol and acetone were acquired from Sinopharm Chemical Reagent Co., Ltd., China. Sodium fluoride (NaF), chromic anhydride (CrO_3_), phosphoric acid (H_3_PO_4_) and boric acid (H_3_BO_3_) were achieved from Shanghai Macklin Biochemical Technology Co., Ltd. Deionized water was generated by Ulupure-II-20T water system in our laboratory.

Material Characterizations

The morphological and elementary composition of the electrodeposited surfaces were performed using a field emission scanning electron microscope (FE-SEM, Hitachi S4800, Japan) equipped with energy dispersive X-ray spectroscopy (EDS). The chemical composition was analyzed by a combination of X-ray photoelectron spectroscopy (XPS, Thermo Scientific K-Alpha) using a corrected spectral line of C1s at 284.6 eV and Fourier transformed infrared spectroscopy (FT-IR, Nicolet IN10, ThermoFisher) with a resolution of 4 cm^-1^. The phase analyses on the thinner electrodeposited surfaces were conducted by a Grazing Incidence X-ray diffractometry (GIXRD, D8 ADVANCE Cu Kα radiation, Bruker) with a fixed incident angle of 0.1°.

The static non-wettability of the electrodeposited surfaces was reflected by CA and CSA, which was measured by a contact angle analyzer (Kruss DSA100, Germany). For all surfaces, at least five repetitions of measurement were performed at independent locations with a water droplet around 6 μL, and the values were statistically averaged to ensure the accuracy of the experimental data.

Molecular Dynamics Simulation Method

The corresponding number of water molecules were set as 9900, 17600, 9870 and 9900 on the plate, A-20, A-30 and A-40 surfaces, respectively. After the atomic coordinates of the substrate are fixed and the system is minimized by energy, kinetic relaxation of 40 ps is performed at the temperature of 298 K to allow liquid water to enter the structure and complete the preparation for modeling. The models are located at the bottom of the simulation box, and a vacuum layer with a height of 400 nm is set above the models, which can completely eliminate the boundary effect of the periodic boundary,^[5]^ as shown in **Figure. S6**.


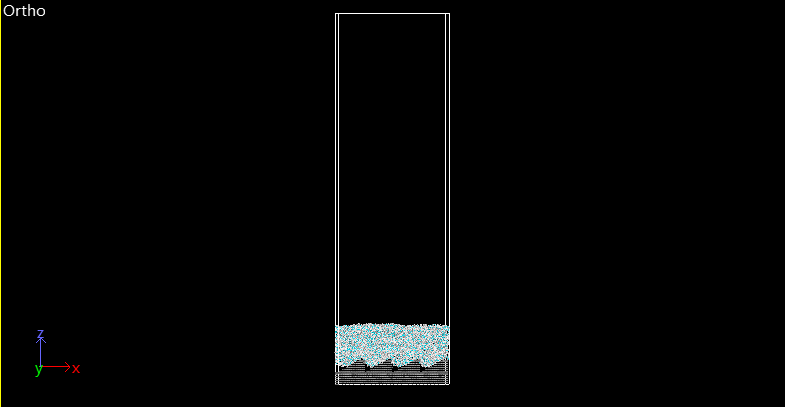


**Figure S6**. The diagram of model setup.

Considering the influence of gravity in the actual freezing environment, the gravity of 6.24×10^-4^ kcal mol^-1^ Angstrom^-1^ was applied in the simulation process. Since the position of the substrate atoms is fixed, there is no need to set up interactions between the substrate atoms. The interaction between water molecules adopts mW coarse-grained potential. The mW is a common using monatomic water model, and the mW model of water does not have hydrogen atoms or electrostatics. Water molecules are represented as a single particle and are able to form tetrahedral “hydrogen-bonded” structures through three-body nonbonded interactions. The interactions between mW water molecules consist of the sum of pairwise and three-body contributions described by the functional form of the Stillinger−Weber (SW) potential:

 (1)

 (2)

 (3)

where *φ_2_* is a two-body term and *φ_3_* is a three-body term. The summations in the equation are over all neighbor atoms of a certain atom within a cutoff distance. In our simulation, it just needs to set up the ε = 6.189 kcal mol^-1^, and σ = 2.3925Å to get mW water model.

Additionally, LJ potential is used to define the force between water molecules and substrates atoms.^[6]^ LJ potential is described by following equation:

$\text{E}=4\varepsilon\left[\left(\frac{\sigma}{r_{ij}}\right)^{12}-\left(\frac{\sigma}{r_{ij}}\right)^{6}\right]$ (4)

Afterwards, to survey the influence of surface energy (*ε_ws_*) variation on icing behavior, different interaction energies are inflicted on the surface. Therein, the contact angles of water droplets are verified under different surface interaction energy conditions, as shown in **Figure. S7**. In this work, the interaction energy of a flat surface is set as 0.12 kcal mol^-1^ so that its contact angle is about 160°, which is similar to the actual contact angle of the flat plate after superhydrophobic treatment for 10 min. Hence, the ε of 0.12 kcal mol^-1^ used to describe the icing behavior on a superhydrophobic surface is considered credible in this work.


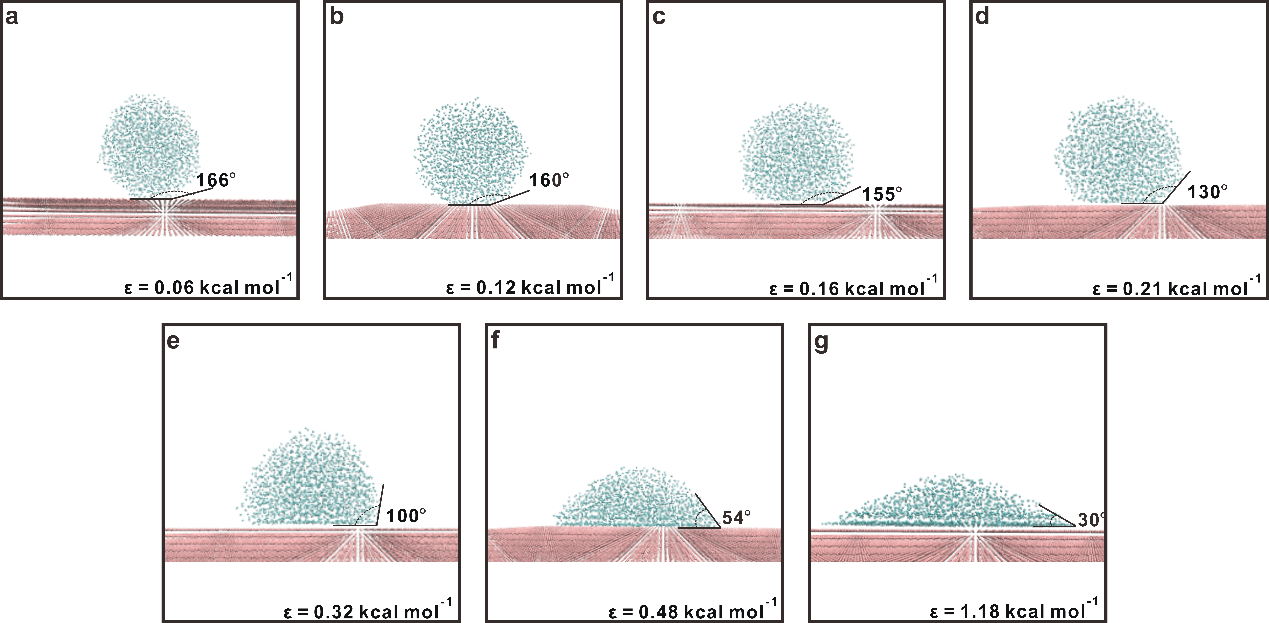


**Figure S7**. Contact angles of plate surface under different interaction energy. a ε=0.06 kcal mol^-1^. b ε=0.12 kcal mol^-1^. c ε=0.16 kcal mol^-1^. d ε=0.21 kcal mol^-1^. e ε=0.32 kcal mol^-1^. f ε=0.48 kcal mol^-1^. g ε=1.18 kcal mol^-1^.

Moreover, three-dimensional periodic boundary conditions were selected for the simulation system. The relevant literature shows that the ramps are performed with cooling rates of 5 K ns^-1^, 2 K ns^-1^, and 1 K ns^-1^, and only the latter resulted in crystallization of ice.^[7]^ Hence, the cooling rate of 1 K ns-1 is adopted in this work. Additionally, the above literature also reveals that the equations of motion of water are integrated with the velocity Verlet algorithm with a time step 5 fs in the case of the systems with an open water/vacuum interface and 10 fs for the bulk systems.^[7]^ Meanwhile, the calculation time step can be extended appropriately due to the employment of coarse granulation potential. On this basis, we believe that a time step of 5 fs is acceptable for this calculation. Hence, the nucleation and growth process were investigated during cooling simulation in which the temperature changed from 290 K to 180 K with cooling rate of 1 K ns^-1^. The Nose method was used to control temperature, which is a suitable cooling rate for observing the nucleation process.^[7]^ Simulations were performed in the NVT ensemble and the equations of water motion were integrated with the velocity Verlet algorithm using a time step of 5 fs.

**References**

1. Wang L, Tian Z, Jiang G, et al. Spontaneous dewetting transitions of droplets during icing & melting cycle. *Nat. Commun*. **2022**, 13, 378.
2. Li N, Jiang J, Yang M Y, et al. Anti-icing mechanism of combined active ethanol spraying and passive surface wettability. *Appl. Therm. Eng*. **2023**, 220, 119805.
3. Maras E, Trushin O, Stukowski A, et al. Global transition path search for dislocation formation in Ge on Si (001). *Comput. Phys. Commun*. **2016**, 205, 13-21.
4. Bi, Y., Cao, B., Li, T. Enhanced heterogeneous ice nucleation by special surface geometry. *Nat. Commun*. **2017**, 8, 15372.
5. Haji-Akbari A, DeFever R S, Sarupria S, et al. Suppression of sub-surface freezing in free-standing thin films of a coarse-grained model of water. *Phys. Chem. Chem. Phys*. **2014**, 16, 25916-25927.
6. M. Fitzner, G. C. Sosso, F. Pietrucci, S. Pipolo, A. Michaelides. Pre-critical fluctuations and what they disclose about heterogeneous crystal nucleation. *Nat. Commun*. **2017**, 8, 2257.
7. Lupi L, Hudait A, Molinero V. Heterogeneous Nucleation of Ice on Carbon Surfaces. *J. Am. Chem. Soc*. **2014**, 136, 3156-3164.
